# Parallelized, Aerobic, Single Carbon-Source Enrichments from Different Natural Environments Contain Divergent Microbial Communities

**DOI:** 10.3389/fmicb.2017.02321

**Published:** 2017-11-28

**Authors:** Theodore M. Flynn, Jason C. Koval, Stephanie M. Greenwald, Sarah M. Owens, Kenneth M. Kemner, Dionysios A. Antonopoulos

**Affiliations:** Biosciences Division, Argonne National Laboratory, Argonne, IL, United States

**Keywords:** microbial ecology, carbon cycling, soil microbiology, microcosm, enrichment culture

## Abstract

Microbial communities that inhabit environments such as soil can contain thousands of distinct taxa, yet little is known about how this diversity is maintained in response to environmental perturbations such as changes in the availability of carbon. By utilizing aerobic substrate arrays to examine the effect of carbon amendment on microbial communities taken from six distinct environments (soil from a temperate prairie and forest, tropical forest soil, subalpine forest soil, and surface water and soil from a palustrine emergent wetland), we examined how carbon amendment and inoculum source shape the composition of the community in each enrichment. Dilute subsamples from each environment were used to inoculate 96-well microtiter plates containing triplicate wells amended with one of 31 carbon sources from six different classes of organic compounds (phenols, polymers, carbohydrates, carboxylic acids, amines, amino acids). After incubating each well aerobically in the dark for 72 h, we analyzed the composition of the microbial communities on the substrate arrays as well as the initial inocula by sequencing 16S rRNA gene amplicons using the Illumina MiSeq platform. Comparisons of alpha and beta diversity in these systems showed that, while the composition of the communities that grow to inhabit the wells in each substrate array diverges sharply from that of the original community in the inoculum, these enrichment communities are still strongly affected by the inoculum source. We found most enrichments were dominated by one or several OTUs most closely related to aerobes or facultative anaerobes from the *Proteobacteria* (e.g., *Pseudomonas, Burkholderia*, and *Ralstonia*) or *Bacteroidetes* (e.g., *Chryseobacterium*). Comparisons within each substrate array based on the class of carbon source further show that the communities inhabiting wells amended with a carbohydrate differ significantly from those enriched with a phenolic compound. Selection therefore seems to play a role in shaping the communities in the substrate arrays, although some stochasticity is also seen whereby several replicate wells within a single substrate array display strongly divergent community compositions. Overall, the use of highly parallel substrate arrays offers a promising path forward to study the response of microbial communities to perturbations in a changing environment.

## Introduction

From the soil under our feet to the deepest sedimentary basins, microbial life inhabits nearly every environment on Earth (Whitman et al., 1998). The abundance and activity of the individual populations that comprise these communities change dynamically in response to changes in their local environment (Nemergut et al., 2013). The composition of microbial communities has been linked to specific parameters like water chemistry in environments such as lakes (Youngblut et al., 2014), streams (Zeglin, 2015), wetlands (Baldwin et al., 2006; Peralta et al., 2010; Dalcin Martins et al., 2017), and aquifers (Flynn et al., 2013; Hug et al., 2015; Kirk et al., 2015). In seawater, for example, the abundance of individual populations of bacteria have been shown to oscillate in sync with changes in light, temperature, and salinity (Eren et al., 2013; Ottesen et al., 2014). Soil, however, possesses such extreme physical, chemical, and biological heterogeneity that understanding the environmental forces that shape the structure and function of microbial communities there remains an outstanding challenge (Tiedje et al., 1999; Roesch et al., 2007; O'Brien et al., 2016; Bailey et al., 2017).

As the number of taxonomic groups within a particular soil often exceeds several thousand distinct clades, there is considerable interest in using simplified microbial communities to test hypotheses related to soil ecology. By “minimizing” a native microbial community from soil or elsewhere through enrichment in the laboratory, noise from the myriad co-existing metabolic networks and structural heterogeneities present in the parent environment can be pared down to focus on a particular process of interest, allowing the power of modern omics technology to be brought to bear on specific questions in microbial ecology (Prosser, 2015). This microcosm approach is frequently used to examine a subset of a native community such as sulfate reducers (Raskin et al., 1996; Kirk et al., 2013; Kwon et al., 2014), denitrifiers (Laverman et al., 2010; Kraft et al., 2014), or organisms capable of degrading specific compounds of interest (Brennan et al., 2006; Sutton et al., 2013; Luo et al., 2014; Onesios-Barry et al., 2014). Conducting such experiments as high-throughput, parallel replicates across a broad variety of environments has the potential to provide greater insight into how microbial communities respond to changing conditions.

Physiological profiling using microtiter plates has been frequently used as a method of examining the functional diversity of mixed microbial communities by monitoring the production of NADH using a redox-active dye (Bochner, 1989; Bochner et al., 2001). This approach allows the utilization of an array of carbon compounds by microbial communities to be monitored in parallel using 96-well plates. These substrate arrays are frequently used to test the metabolic capabilities of the microbiome inhabiting soil, water, and other environments (Bartscht et al., 1999; King, 2003; Weber and Legge, 2009; Gryta et al., 2014; Zhang Y. et al., 2014). Given that the composition of the communities that grow from the inocula in these arrays is rarely, if ever, characterized directly, the extent to which the “active” populations utilizing a particular substrate are representative of the community at large remains unclear (Konopka et al., 1998; Haack et al., 2004). Substrate arrays also offer an opportunity to study in parallel the response of a single inoculum to the addition of a wide array of nutrients.

In this study, we employed these substrate arrays as mini-bioreactors to monitor the response of microbial communities from six distinct environments to enrichment on 31 individual carbon compounds. We hypothesize that the fractionation of the complex natural communities will be more influenced by the nature of the carbon source utilized and select for a specific subset of microorganism(s) regardless of source environment. To this end, we selected as inocula soil, water and sediment from a variety of terrestrial environments including a wetland, a grassland, and three types of forest (temperate, subalpine, and tropical). We sought to test (A) the extent to which communities enriched in these substrate arrays are representative of the initial community as a whole, (B) the degree to which the initial structure of a community determines its response to a press disturbance (increase in nutrient levels) for different classes of environmentally-relevant (Hitzl et al., 1997) carbon compounds and (C) whether the composition of the communities that grow to inhabit the substrate arrays are more influenced by the composition of the inoculum or the type of carbon source upon which they are enriched.

## Materials and methods

### Experimental setup

Substrate array experiments were conducted by inoculating 96-well plates with soil suspensions or natural waters from six distinct environments. For these substrate arrays we chose the EcoPlate array (Biolog, Hayward, CA). Each well of this substrate array contains a proprietary minimal media (see Bochner et al., 2001) as well as one of 31 carbon sources representing five distinct categories: amines, amino acids, carbohydrates, polymers, and phenolic compounds (Table 1). While an exact formulation of the media is not publicly available, the manufacturer's documentation states that medium components are present at concentration of 2–20 mM for the carbon source, 1–5 mM for N, 0.1–1 mM for P, 0.1–1 mM for S, and <2 μM of a vitamin solution. A tetrazolium dye that reacts irreversibly with NADH is also present in each well, forming a distinct purple color in the presence of actively respiring cells. The intensity of this color change is proportional to the amount of NADH produced and was quantified by measuring absorption at 592 nm using a plate reader to approximate the level of metabolic activity (Bochner, 1989).

**Table 1:** List of 31 carbon substrates present in substrate arrays (one substrate per well, three replicate wells per substrate).

**CS number**	**Carbon source**	**Category**
1	2-Hydroxybenzoate	Phenol
2	4-Hydroxybenzoate	Phenol
3	N-acetyl-D-glucosamine	Carbohydrate
4	α-Cyclodextrin	Polymer
5	α-Keto butyric acid	Carboxylic acid
6	Arginine	Amino acid
7	L-asparagine	Amino acid
8	β-Methyl-D-glucoside	Carbohydrate
9	Cellobiose	Carbohydrate
10	I-Erythritol	Carbohydrate
11	D-galactonic acid lactone	Carbohydrate
12	D-galacturonic acid	Carboxylic acid
13	γ-Hydroxybutyric acid	Carboxylic acid
14	D-glucosaminic acid	Carboxylic acid
15	Glucose-1-phosphate	Carbohydrate
16	D,L-α-glycerol phosphate	Carbohydrate
17	Glycogen	Polymer
18	Glycyl-L-glutamic acid	Amino acid
19	Itaconic acid	Carboxylic acid
20	α-D-lactose	Carbohydrate
21	D-malic acid	Carboxylic acid
22	D-mannitol	Carbohydrate
23	L-phenylalanine	Amino acid
24	Phenyl ethylamine	Amine
25	Putrescine	Amine
26	Methyl pyruvate	Carboxylic acid
27	L-serine	Amino acid
28	L-threonine	Amino acid
29	Tween 40	Polymer
30	Tween 80	Polymer
31	Xylose	Carbohydrate

Given the spatial heterogeneity that can exist in even closely adjacent soil environments (O'Brien et al., 2016), we chose as inocula samples collected across several scales of geographic separation. Soil from a temperate climate was collected from a restored prairie grassland (FLP) and an adjacent forest (FLF) on site at the Fermi National Accelerator Laboratory (Fermilab) in Batavia, Illinois, USA (41.842888, −88.264180). Surface water (AWW) and soil (AWS) was obtained from a palustrine emergent wetland populated by *Typha* and *Phragmites* spp. on site at Argonne National Laboratory in Lemont, Illinois, USA (41.710022, −87.986241). Tropical forest soil (CRP14) was obtained from a Caribbean lowland rainforest (10.183333, −84.666667) within the EARTH University Forest Reserve in Costa Rica (Alvarez-Clare et al., 2013). Subalpine forest soil (SodaSpr) was taken from a pine forest near Soda Springs, California, USA (39.306120, −120.381295) at an elevation of 2,063 m. Soil samples were taken during the summer by removing ~100 g of soil to a depth of 5 cm with a clean, ethanol-sterilized hand trowel after clearing off any surface litter. Aquatic samples were taken at the water's surface using a sterile, 1 L container. Samples were used to inoculate substrate arrays within 72 h of collection to minimize any potential long-term storage effects.

**Figure 1:**
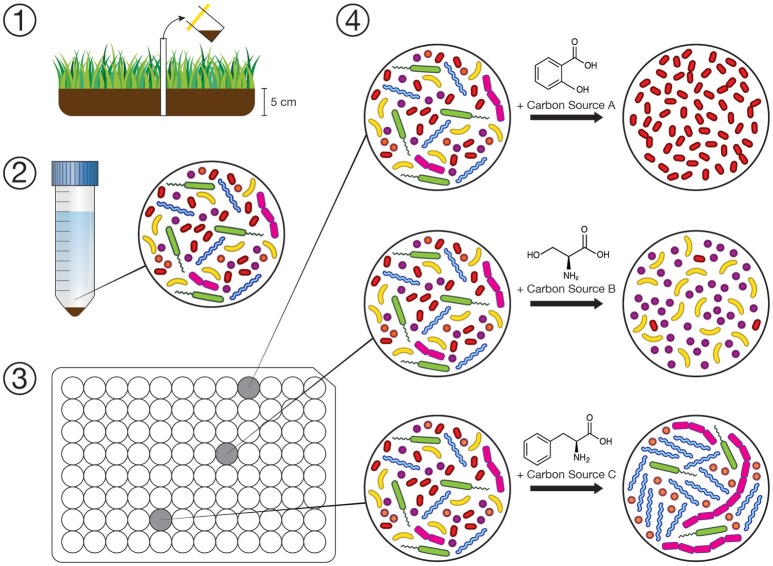
Conceptual diagram of our experimental workflow. (1) Collection of inocula for substrate arrays. (2) Dilute suspension of soil/water microbes prepared within 72 h. (3) Substrate arrays where each well is amended with one of 31 individual carbon sources (in triplicate), inoculated with suspension (1:100 m/v dilution), and incubated for 72 h. (4) Changes in composition of microbial communities in the substrate array is assayed by sequencing 16S rRNA gene amplicons on the Illumina MiSeq platform.

A conceptual diagram of our experimental design is shown in Figure 1. Solid suspensions used to inoculate the bioreactors were prepared by adding 1 g of soil to 5 mL of sterile, DNA-free ultrapure water (HyClone HyPure molecular grade, Thermo Scientific). Suspensions were then homogenized using an ultrasonic dismembrator at low intensity for 30 s, breaking up any aggregates and removing bacteria from organic and mineral particles. The slurry was then diluted 1:100 (v/v) by adding additional sterile water and dispensed into a substrate array (150 μL per well). When using an aqueous medium as an inoculum in the case of the wetland surface water, the inoculum was amended directly to the array without dilution. Triplicate subsamples of the inocula were set aside and frozen immediately at −80°C. After 72 h of incubation at 30°C, 100 μL aliquots were taken from each well of the substrate array and stored at −80°C until analyzed by sequencing 16S rRNA gene amplicons.

### Molecular microbiology and bioinformatics

DNA was extracted from both environmental inocula and aliquots from the substrate arrays using MO BIO PowerSoil kits following the manufacturer's instructions. The concentration of DNA extracted was quantified using Quant-iT PicoGreen dsDNA assay (Invitrogen). 16S rRNA genes from each of these samples were amplified by the polymerase chain reaction (PCR) using a primer set (515F-806R) targeting the V4 region of this gene in both bacteria and archaea (Bates et al., 2011; Caporaso et al., 2011). These primers were barcoded to allow for sample multiplexing on the Illumina MiSeq (Caporaso et al., 2012). PCR reactions were carried out using 5 PRIME MasterMix (Gaithersburg, MD). PCR conditions used an initial denaturation step of 95°C for 3 min followed by 35 cycles of 95°C for 30 s, 55°C for 45 s, then 72°C for 1.5 min and finalized by a single extension step 72°C for 10 min. Pooled product for each sample was then quantified using the PicoGreen assay. Primer dimers were removed using the UltraClean PCR Clean-Up Kit (MO BIO Laboratories, Inc.) and the amount of DNA in each sample was normalized to a final concentration of 2 ng μL^−1^.

Paired-end amplicons (151 × 12 × 151 bp) were sequenced on an Illumina MiSeq at the Environmental Sample Preparation and Sequencing Facility at Argonne National Laboratory following procedures described in Caporaso et al. (2012). In all cases, sequencing workflows included both experimental and PCR/library preparation control blanks that failed to generate sequencing libraries of sufficient quality to provide any usable sequences. These controls were put through the same sequencing pipeline as the samples, but consistently failed to generate usable sequences. Any apparent sequences that were produced from these controls were all eliminated during the sequence screening process and likely arose from the formation of primer dimers or mispriming, rather than contamination.

A total of 19.0 million paired-end sequences were generated from 594 samples (576 substrate array samples and six triplicate inocula samples) for a depth of 32,552 ± 20,696 sequences per amplicon library. Paired-end reads were joined using PEAR (Zhang J. et al., 2014), and combined libraries were processed together using QIIME version 1.9.1 (Caporaso et al., 2010b). Poor-quality sequences were discarded based on Phred score, primer mismatches, divergence from expected amplicon length (253 base pairs) using the default settings in QIIME (split_libraries_fastq.py). Sequences were clustered into *de novo* operational taxonomic units (OTUs) at 97% similarity using QIIME's pick_de_novo_otus.py command. Singleton OTUs (defined as OTUs represented by only a single representative sequence across all samples) were discarded. Representative sequences from each OTU were aligned using PyNAST (Caporaso et al., 2010a) and classified according to the Greengenes taxonomy (version gg_13_8; McDonald et al., 2012). A phylogenetic tree of representative sequences was constructed with FastTree (Price et al., 2010) and used to calculate UniFrac distances between each sample as a measure of beta diversity (Hamady et al., 2009; Lozupone et al., 2011). Beta diversity was visualized in R using non-metric multidimensional scaling (NMDS) using the phyloseq package (McMurdie and Holmes, 2013). Analysis of similarity (ANOSIM) was calculated between groups of samples using Primer-7 (Clarke and Warwick, 2001). The differential abundance of specific OTUs was calculated in R using the package edgeR (Robinson et al., 2010) implemented in phyloseq. Significance testing for edgeR was done with tagwise tests using the exact negative binomial test (Robinson and Smyth, 2008) and a False Discovery Rate (FDR; Benjamini-Hochberg) significance threshold of 0.05 (Benjamini and Hochberg, 1995). Alpha diversity calculations (Shannon index of diversity) for each sample were made in R using phyloseq. Raw sequence data is freely available to download over the internet through the Argonne Scientific Publications portal and can be accessed using the Digital Object Identifier (DOI) 10.17038/1371460.

## Results

**Figure 2:**
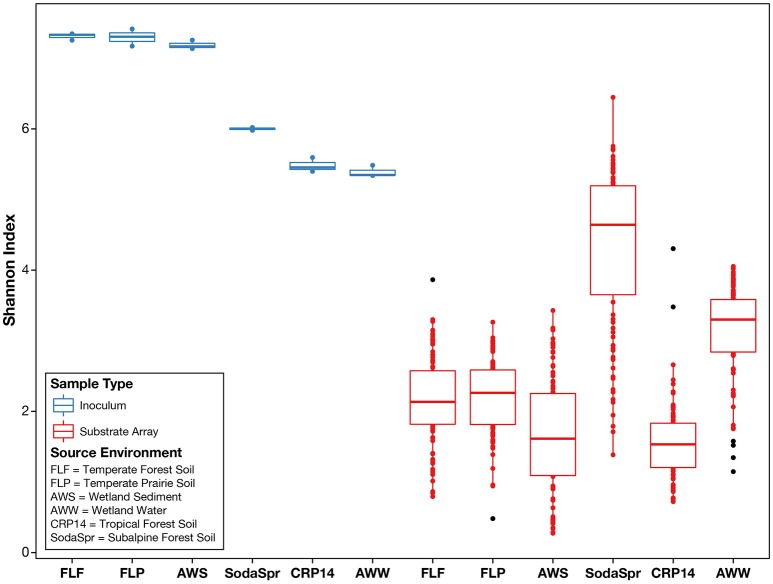
Alpha diversity of environmental inocula and EcoPlate enrichments as measured by the Shannon index of diversity. On average, the total diversity of environmental communities decreases substantially following incubation in the substrate array, regardless of carbon source.

We observed that, following incubation for 72 h at 30°C, the structures of the microbial communities in the wells of each substrate array deviated sharply from the initial composition of the inoculum. As shown in Figure 2, the average alpha diversity as measured by the Shannon index decreased sharply in the enrichments of all six substrate arrays, falling substantially below the average diversity of the inocula. Comparisons using the Mann-Whitney *U*-test between the Shannon indices of the inoculum samples and those of the substrate array samples for each environment showed a significant decrease (*P* < 0.005) in diversity for all six environments [AWS (0.003); AWW (0.003); CRP14 (0.003); FLF (0.003); FLP (0.003); SodaSpr (0.004)].

**Figure 3:**
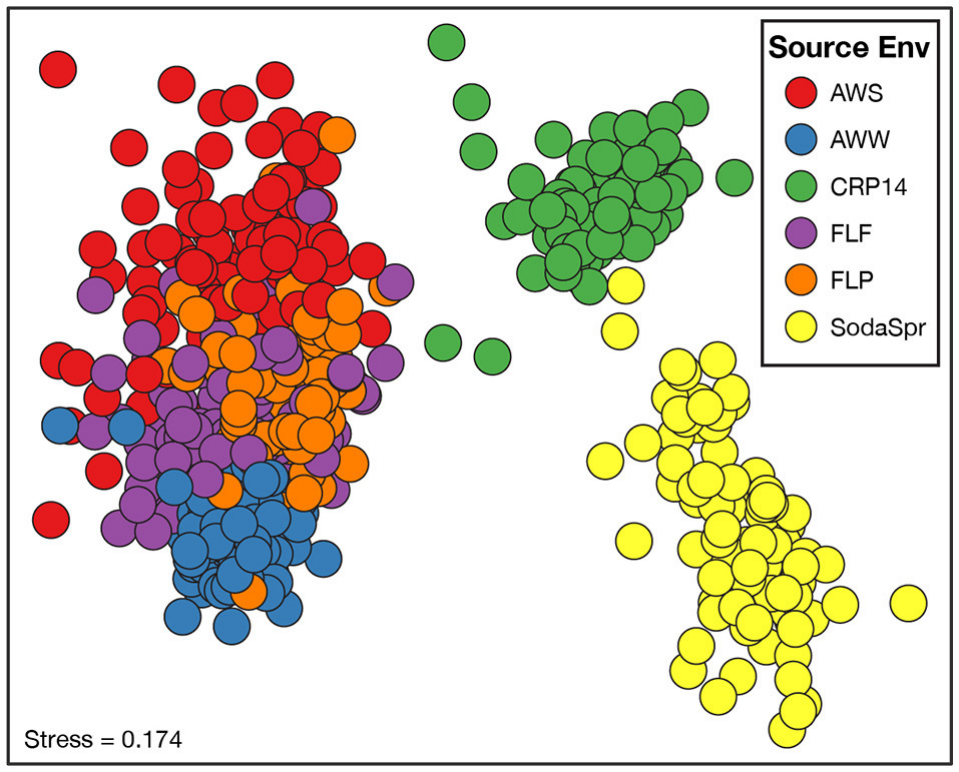
Non-metric multidimensional scaling (NMDS) analysis based on the weighted UniFrac distance metric showing the difference in microbial community structure between the substrate array enrichments.

Beta diversity comparisons using NMDS analysis found large differences in the composition of these communities compared to the inoculum based on the weighted UniFrac distance (Figure S1). While the microbial communities in each well of the substrate array diverged from the parent material, they differed substantially from one another as well (Figure 3). The degree of differences between substrate arrays largely recapitulate the observed differences between inocula (Figure S2), with one notable exception. The most divergent communities are from substrate arrays inoculated with soil from the SodaSpr and CRP14 sites. These enrichments differ substantially both from the arrays inoculated with material from temperate environments (AWW, AWS, FLF, and FLP) and from each other. Within those inoculated with materials from Illinois, the FLF and FLP substrate array communities overlap almost entirely. AWS and AWW communities are more separated from each other, but still cluster together with the enrichments inoculated with other Illinois materials (Figure 3). Comparing measurements of growth (qPCR quantification of 16S rRNA gene copies) and activity (color development of NADH-reactive tetrazolium dye) showed a generally positive trend between the two in the substrate arrays but no relationship was observed between either measurement and community composition (data not shown).

**Figure 4:**
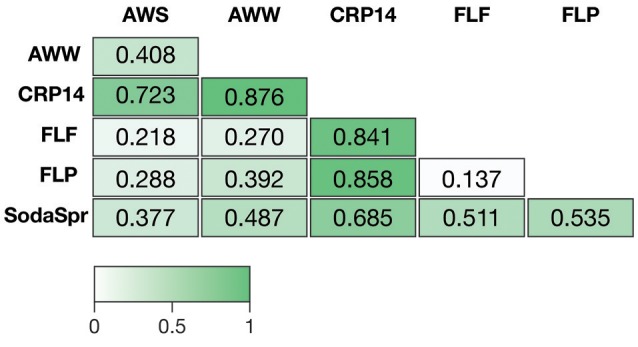
Analysis of similarity (ANOSIM) R-values showing differences in microbial community composition between each of the six substrate arrays. The value of *p* for all comparisons was <0.0001%.

Based on ANOSIM shown in Figure 4, the clustering by environment observed in the NMDS ordination shown in Figure 3 is statistically significant for pairwise comparisons between nearly all six substrate arrays. The CRP14 communities differ the most from the other substrate arrays, with R-values (R_ANOSIM_) ranging from 0.685 to 0.876 (*p* < 0.0001%). An R_ANOSIM_ > 0.75 indicates two groups of microbial communities are almost entirely distinct, while a value between 0.25 and 0.75 indicates some degree of overlap (Ramette, 2007). Values of R_ANOSIM_ < 0.25 indicate the two groups of communities being compared are considered barely separable. Based on this metric, CRP14 enrichments are most similar to (although still mostly distinct from) the SodaSpr enrichments (R_ANOSIM_ = 0.685, *p* < 0.0001%). SodaSpr enrichments fall on a gradient of distinction between the tropical and temperate enrichments (R_ANOSIM_ = 0.377–0.685, *p* < 0.0001%), while the temperate soil enrichments show almost complete overlap with one another (R_ANOSIM_ = 0.136–0.288, *p* < 0.0001%). The two wetland substrate arrays (AWW and AWS) are distinct from one another but share some overlap (R_ANOSIM_ = 0.408, *p* < 0.0001%).

**Figure 5:**
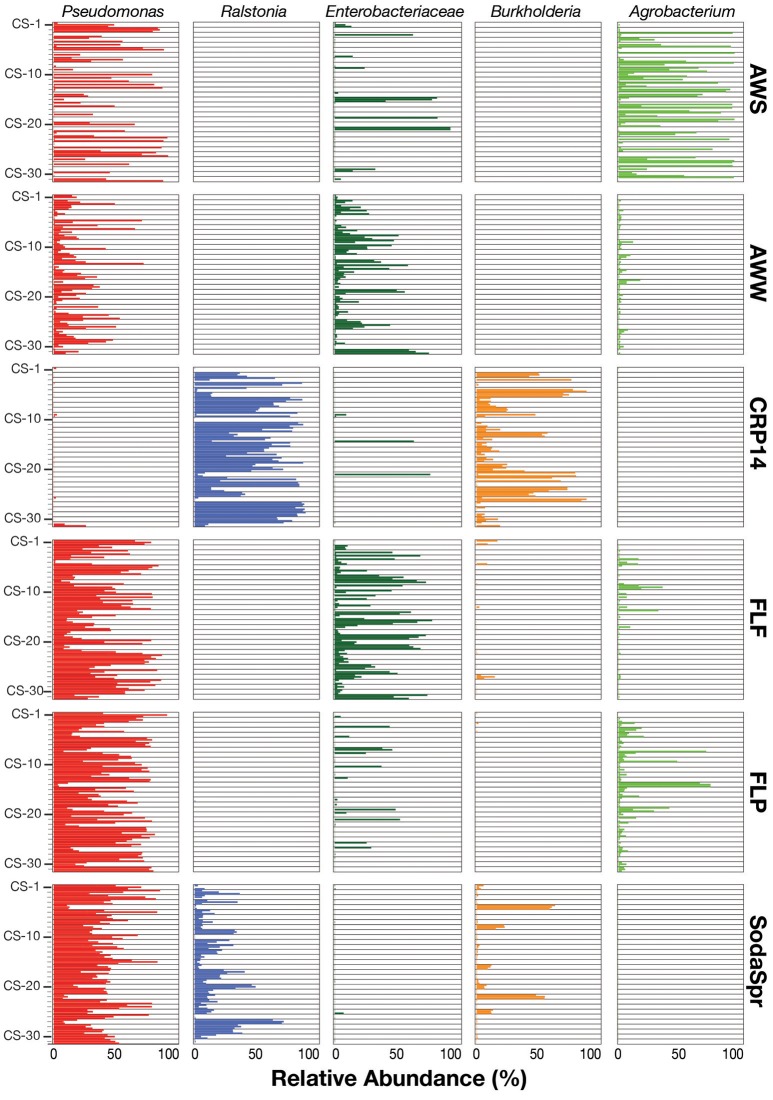
Relative abundance of the top five taxa (*Pseudomonas, Ralstonia, Agrobacterium, Burkholderia*, and *Enterobacteriaceae*) in the EcoPlate enrichments, with triplicate samples grouped by carbon amendment.

Inspection of the most abundant organisms in each substrate arrays reveals that many of the enrichments in each substrate array are inhabited by one or several dominant taxa (Figure 5). These taxa, of which OTUs classified as *Pseudomonas, Ralstonia*, the family (fa.) *Enterobacteriaceae, Burkholderia*, and *Agrobacterium* are the most abundant, are not the most abundant taxa in the inocula. Sequences classified in the genus *Pseudomonas* account for 36% of all sequences across all six substrate arrays, but have an average abundance of only 0.26 ± 0.17% in the inocula. In five of the six substrate arrays (except CRP14), the relative abundance of *Pseudomonas* is on average 61–300 fold greater relative to the inoculum. In CRP14 *Pseudomonas* was only enriched four-fold, on average, and comprised <0.3% of the total diversity seen across all wells and was of >1% abundance in only three wells (3−11% in those wells). Others, like *Ralstonia*, fa. *Enterobacteriaceae, Burkholderia*, and *Agrobacterium* are considerably enriched in many if not most of the wells within individual substrate arrays, but are largely or entirely absent in others (Figure 5).

**Figure 6:**
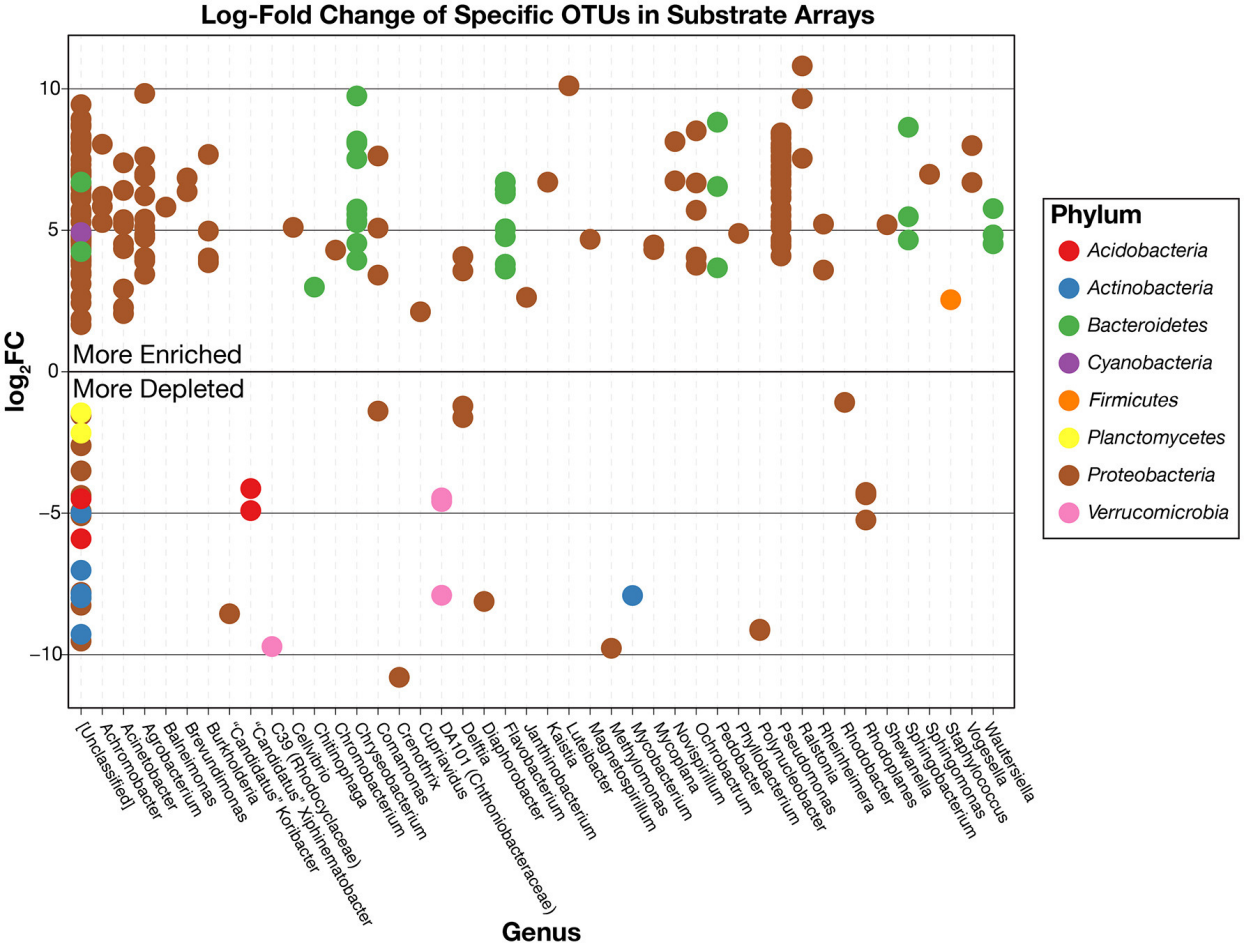
Log2-fold change in the relative abundance of individual OTUs in the substrate array enrichments relative to the original inoculum based on the edgeR package. Positively enriched OTUs are more abundant, on average, in the substrate arrays than in the inocula while negatively enriched OTUs are more abundant in the inocula. OTUs are separated horizontally by genus and colored by phylum.

**Figure 7:**
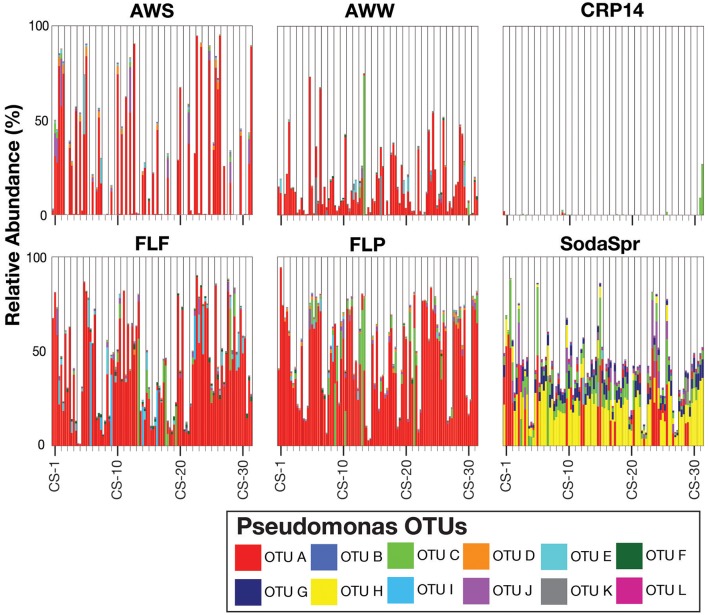
Relative abundance of individual OTUs classified within the genus *Pseudomonas*.

This pattern is also true for less broadly abundant taxa like *Comamonas* and the fa. *Xanthomonadaceae*. The distribution of all OTUs enriched or depleted (based on edgeR) in the substrate arrays compared to the inocula is shown in Figure 6. Overall, these data confirm the observational data shown in Figure 5, that the OTUs most abundant in the enrichments are largely members of the *Proteobacteria* (*Pseudomonas, Ralstonia, Agrobacterium*) and the *Bacteroidetes* (*Chryseobacterium, Flavobacterium, Sphingobacterium, Pedobacter*, and *Wautersiella*). OTUs classified as other phyla (e.g., *Actinobacteria, Verrucomicrobia*, and *Acidobacteria*) are considerably less abundant in the enrichments than they are in the original environments. The distribution of specific OTUs also varies within taxonomic groups. As seen in Figure 7, in four of the five substrate arrays where *Pseudomonas* is abundant (AWS, AWW, FLF, and FLP), a single OTU predominates (labeled OTU A). While OTU A is present in some enrichments inoculated with material from the SodaSpr site, a different OTU (OTU H) is much more dominant. Overall, of the 10 taxonomic groups that were found to be above 1% average abundance in the substrate arrays (*Pseudomonas, Ralstonia*, fa. *Enterobacteriaceae, Agrobacterium, Burkholderia*, fa. *Aeromonadaceae, Chryseobacterium*, fa. *Xanthomonadaceae, Delftia, Comamonas*), sequences from all these together comprise only 1.1–2.5% of the total diversity of the inocula in the five soil arrays and 8.0% of the diversity in the wetland water array.

Additional pairwise ANOSIM calculations were conducted to test for systematic differences in community composition between enrichments amended with a specific substrate category (amines, amino acids, carbohydrates, polymers, and phenolic compounds) and within each individual substrate itself (Supplementary Table 1). This test compared whether communities from across all six substrate arrays amended with a specific class of carbon compound (e.g., amines) were, on average, more similar to each other than to communities amended with a different class of compounds (e.g., carbohydrates). While no statistically significant correlations held observed across all six substrate arrays, several statistically significant ANOSIM-values were observed for several substrate categories within a given environment. Most significantly, comparing enrichments amended with a carbohydrate with those amended with a phenolic compound yield statistically significant ANOSIM values (R_ANOSIM_ = 0.483–0.597) in three of the six environmental enrichments: wetland water, temperate forest soil, and subalpine forest soil. None of the other within-environment comparisons yielded significant results for more than one of the six environmental sources.

**Figure 8:**
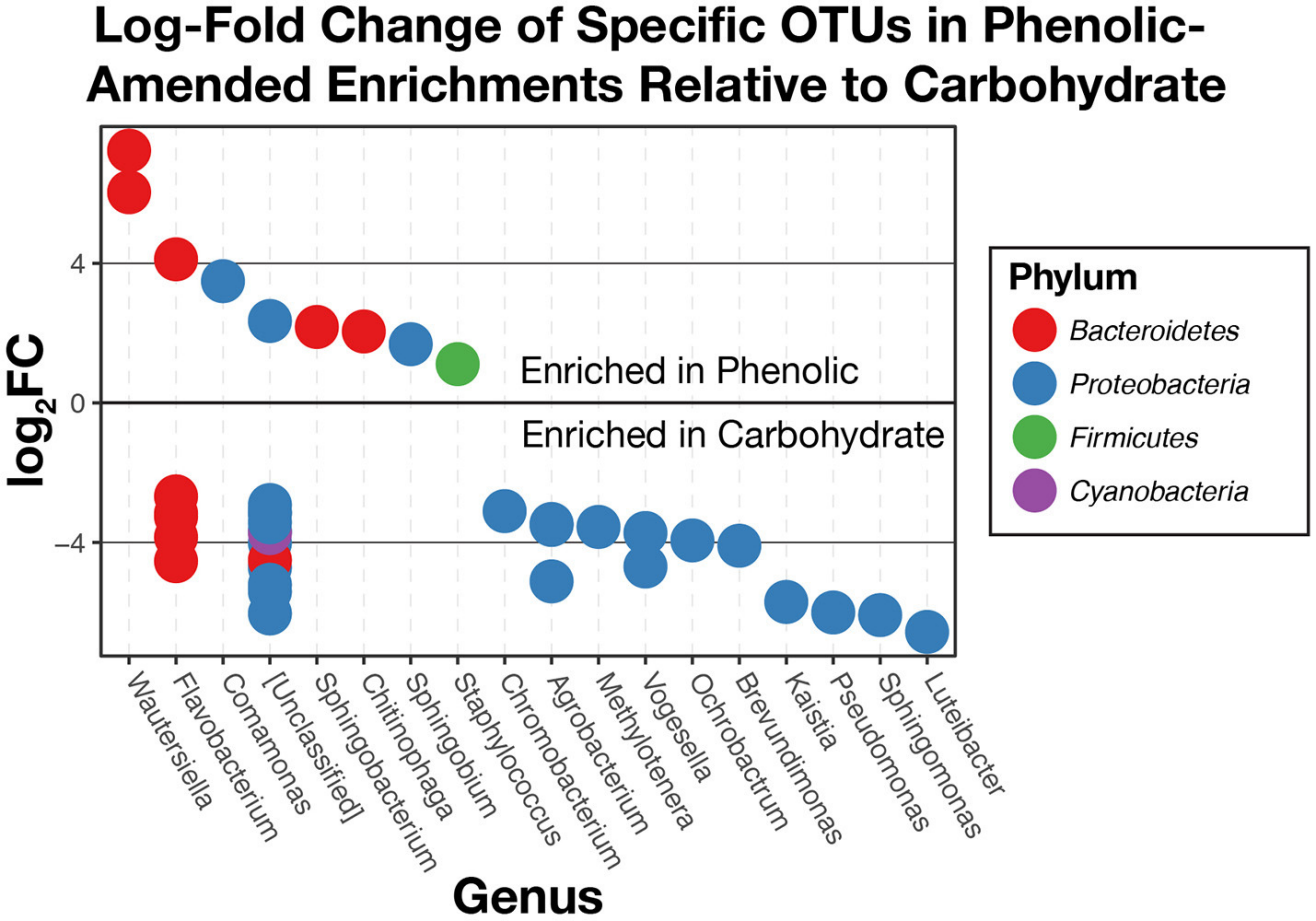
Log2-fold changes of differentially abundant operational taxonomic units (OTUs) in substrate arrays wells given phenolic substrates compared to carbohydrates. OTUs that are positively enriched are more abundant in phenolic enrichments. Those that are negatively enriched are more abundant in carbohydrate enrichments.

Additional comparisons between phenolic compound-amended and carbohydrate-amended enrichments made using edgeR found that 42 OTUs above the chosen total variance threshold of 10^−5^ (McMurdie and Holmes, 2013) were differentially abundant (Figure 8). Overall, we found that OTUs within the phylum *Bacteroidetes* were generally more abundant in the phenolic-amended enrichments, while OTUs classified within the *Proteobacteria* were generally more abundant in carbohydrate-amended enrichments. OTUs classified in the genera *Wautersiella, Comamonas, Sphingobacterium, Chitinophaga, Sphingobium*, and *Staphylococcus* were more abundant in the phenolic-amended microcosms.

## Discussion

The distinct communities of microorganisms that grow to dominate each enrichment within the substrate arrays bear the strong influence of the initial composition of the soil or water used as inoculum. As can be seen from the NMDS plot in Figure 3, the source of inoculum is by far the strongest signal in differentiating the microbial communities in these substrate arrays. The statistical significance of this result is confirmed by ANOSIM based on weighted UniFrac^1^, where the resulting R-values recapitulate the trends observed in the NMDS plot (Figure 4). Furthermore, these results show that, for the most part, the communities in the substrate arrays from the most divergent inocula [tropical (CRP14) and subalpine (SodaSpr) forest soil] remain divergent from those sourced from temperate soil inocula (AWS, FLP, FLF), which beta diversity comparisons indicate are more similar to each other (Figure S2). The exception is the wetland surface water (AWW), which Figure S2 shows has the most divergent community composition of the six inocula prior to enrichment. Following the 72-h incubation, however, the communities that arise in the substrate arrays inoculated with AWW material appear much more similar to the arrays inoculated with temperate soil than to those inoculated with soil from CRP14 or SodaSpr (Figure 3). This suggests that the generalists like *Pseudomonas*, fa. *Enterobacteriaceae*, and *Agrobacterium* that grow to dominate the substrate arrays share a similar baseline activity across all the temperate environments and are therefore able to be enriched to a similar degree in the arrays. Still, the smaller but still significant R_ANOSIM_-values between some of the terrestrial enrichments (e.g., AWW and AWS) indicate the existence of smaller but significant differences between these environments.

Despite the strong influence of the initial inoculum, the overall structure of the microbial communities that grow to inhabit these substrate arrays bears little resemblance to that of the parent material, as measured by both alpha (Figure 2) and beta diversity (Figure S1). In most cases, the enrichments in each substrate array are primarily dominated by one or several OTUs most closely related to aerobic, heterotrophic generalists like *Pseudomonas, Burkholderia, Ralstonia*, fa. *Enterobacteriaceae*, and others (Figures 5, 6). *Pseudomonas* in particular dominated five of the six substrate arrays, although it was nearly absent from enrichments in the array inoculated with soil from site CRP14. *Pseudomonas* and its relatives are well-known as metabolic generalists and are frequently found in aerobic soil environments. Initially, “pseudomonads” were defined largely by “their most striking and ecologically significant group character… namely, the ability to use a wide variety of organic compounds as carbon and energy sources for aerobic growth” (Stanier et al., 1966). Numerous microcosm and enrichment studies from soils under aerobic conditions have found abundant growth of *Pseudomonas* (Greene et al., 2000; Eriksson et al., 2003; Ma et al., 2006), although these are often limited in scope to a few replicates or the utilization of a particular compound of interest.

An exception to this repeated occurrence of *Pseudomonas* was in the apparent swapping of *Pseudomonas* for *Burkholderia* and *Ralstonia* across the CRP14-inoculated enrichments. Like *Pseudomonas, Burkholderia* is a metabolic generalist well-distributed in soil environments (Cho and Tiedje, 2000; Salles et al., 2004; Janssen, 2006; Compant et al., 2008; Lauber et al., 2009; Stopnisek et al., 2014). *Ralstonia* also is a genus commonly found in soil environments (Janssen, 2006), although most studies of this genus focus on its role as soil-borne plant pathogen (Castillo and Greenberg, 2007; Leonard et al., 2017) with a wide distribution in warm and tropical climates (Genin and Boucher, 2004). Both *Burkholderia* and *Ralstonia* share many characteristics with *Pseudomonas*; both were at one time themselves classified as part of that genus (Yabuuchi et al., 1992, 1995) although they are now recognized as *Betaproteobacteria* while *Pseudomonas* is classified as a member of the *Gammaproteobacteria*. The abundance of these two genera in the CRP14-amended substrate array suggests an optimization of the community from the tropical forest system to the set of niches found in the source environment to the exclusion of *Pseudomonas*. There is likely some consistent factor associated with those niches, however, so that a “distinct generalist” is enriched consistently over time in one environment but not another.

When comparing the substrate array enrichments by the chemical class of substrate (amines, amino acids, carbohydrates, polymers, or phenolic compounds) rather than source environment, we found that significant differences in community composition exist primarily between those wells given a carbohydrate as a carbon source and those enriched with a phenolic compound (Figure 8). These differences were observed across three of the six environmental inocula: AWW, FLF, and SodaSpr, while none of the other pairwise comparisons between carbon source class showed a significant difference in more than one substrate array. Taken together, these results suggest that the co-occurrence of minimized community members within these three environments and grown on carbohydrates or phenolics are likely non-random associations. Like the most abundant taxa across the substrate arrays, most of the differentially-abundant OTUs (Figure 8) between phenolic- and carbohydrate-amended enrichments are most closely related to aerobic heterotrophs. Interestingly, the closest neighbors of several of the OTUs that are differentially abundant in phenolic-amended enrichments are organisms that are specifically known for degrading aromatic compounds. For example, members of the genus *Comamonas* have previously been isolated and studied based on their ability to degrade polycyclic aromatic hydrocarbons (Goyal and Zylstra, 1996) while isolated members of the genus *Sphingobium* are known for the ability to degrade phenolic compounds such as nonylphenols (Ushiba et al., 2003) and chlorophenols (Cai and Xun, 2002) as well as other aromatic hydrocarbons (Cunliffe and Kertesz, 2006). Carbohydrate-amended enrichments were more enriched with OTUs classified as *Luteibacter, Sphingomonas, Pseudomonas, Kaistia, Brevundimonas, Ochrobactrum, Vogesella*, and others. Unsurprisingly, known isolates from these groups are primarily aerobic heterotrophs with wide metabolic diversity, including the ability to grow on carbohydrates as an energy source (Lessie and Phibbs, 1984; Lebuhn et al., 2000; Im et al., 2004; de Boer et al., 2007) and are commonly found in a variety of terrestrial environments (Fredrickson et al., 1999; Cho and Tiedje, 2000; Spiers et al., 2000; Lauber et al., 2009). Despite being more enriched in carbohydrate-amended wells, *Sphingomonas* spp. have also shown the ability to grow on aromatic compounds (Fredrickson et al., 1995).

Taken together, these findings provide substantial insight into how microbial communities respond to changes in nutrient conditions. Microbial communities, like all communities of living organisms, are thought to be shaped by two types of ecological factors: those that are more deterministic are dubbed “niche” while those that are more stochastic are “neutral” (Vellend, 2010; Stegen et al., 2012). Niche factors that influence microbial community dynamics include environmental factors such as temperature or pH, the presence or absence of a particular nutrient (e.g., carbon, nitrogen, phosphorus, etc.) or the physical structure of the environment itself (e.g., the porosity or mineralogy of the soil matrix). Neutral forces, conversely, include random birth/death events, migrations of a particular population from one area to another, and other probabilistic events (Chase and Myers, 2011; Stegen et al., 2013). Evidence of the significance of both processes in shaping microbial communities has been observed throughout a variety of environments including soil, seawater, and aquifers (Gilbert et al., 2012; Shade et al., 2012; Handley et al., 2014; Graham et al., 2017). Understanding the relative importance of these factors is of particular importance in microbial ecosystems, where physiologically-relevant gradients can occur on the scale of micrometers. This heterogeneity at the microscale can be found in biofilms (Battin et al., 2016), soil pores (Bailey et al., 2013, 2017), and other sedimentary environments (Jakobsen, 2007), potentially imposing both niche and dispersal limitations at scales far smaller than that of the typical environmental sample. This poses a significant challenge to creating phylogenetically-resolved predictive models of microbial activity (Graham et al., 2016), particularly considering the breathtaking diversity of natural microbial communities (Howe et al., 2014; Hug et al., 2016).

While these experiments were not designed to explicitly test the extent to which niche and neutral factors lead to the composition of microbial communities within the substrate arrays, we see qualitative evidence of both processes shaping the overall composition of these communities. The degree to which either of these two processes influences the resultant minimized community, however, likely depends on the class of substrate. For example, niche selection clearly determines the overall trajectory of the response of the initial inoculum to changes in environmental conditions, as the organisms that grow to dominate the wells of the substrate arrays are aerobic heterotrophs, matching the oxic and carbon-rich conditions found in the wells. Further, niche selection appears evident in the distinction between carbohydrate- and phenolic-amended enrichments, as specific populations closely related to isolates known for degrading aromatic compounds become significantly more abundant in the presence of phenolic compounds (Figure 8).

Still, despite the fact that the source of inoculum is a significant determinant of the overall community structure (Figure 3), probabilistic events still appear to control some of the finer aspects of the composition of the substrate array communities. The extent to which triplicate wells on the same substrate array ended up with the same community composition varied depending on both the substrate and the population. For example, when populations classified as *Burkholderia* became abundant in wells inoculated with soil from the SodaSpr site, the abundance was observed uniformly across all three replicates (Figure 6). Conversely for some of carbon sources in wells amended with AWS soil, two of the three triplicates contained >90% *Pseudomonas* while the third well was similarly dominated by sequences classified as either fa. *Enterobacteriaceae* or *Agrobacterium* (Figure 6). Because the inocula were rigorously diluted, dispersed, and homogenized prior to being added to the substrate arrays, it appears unlikely that this is an artifact of sample preparation. Instead, stochasticity and imperceptible differences in the initial inoculum likely play at least some role in determining the overall trajectory of change in community composition. This has been seen elsewhere, as in Kwon et al.'s (2016) observation of two distinct mineralogical and microbiological trajectories in replicate microcosms amended with glucose and ferric iron.

The extent to which the communities within the substrate arrays represent the environments from which they were enriched depends upon how one looks at the question. In one sense, the communities in the substrate arrays retain components (populations) of the initial community as evidenced by the clustering by environment observed in Figure 3. However, the composition of these communities bears little resemble to that of the source inoculum when compared by measures of alpha (Figure 2) and beta (Figure S1) diversity. This is likely primarily due to the differences in the physicochemical makeup within the substrate arrays compared to that of the source environments. Whereas, soil and sedimentary environments display a complex, heterogeneous three-dimensional structure with dynamic changes in temperature, nutrient availability, and other variables, the substrate arrays are relatively homogeneous in comparison. In fact, the substrate arrays that shared the most taxa with their initial inoculum were those inoculated with wetland surface water, where shared taxa comprised 8.0% of the inoculum diversity compared to 1.1–2.5% in the five substrate arrays inoculated with soil suspensions. While still different in many respects, the wetland surface water does most closely resemble the aqueous environment of the substrate array wells.

The relative physical homogeneity of the substrate arrays compared to the source environments might be one explanation for the rapid dominance of one or few taxa in most of the substrate arrays over the 72-h incubation period. Previous work has shown that physical structure in laboratory microcosms enhances the stable coexistence of multiple interacting soil bacteria (Kim et al., 2008). Other work, however, has found many co-existing microbial populations can exist stably in homogeneous liquid medium (Kraft et al., 2014; Sutton et al., 2015; Chen et al., 2017). For example, recent work has shown that found that co-existence of bacterial isolates could be tied to mortality under different growth conditions within the same media (Friedman et al., 2017). While the goal of our experiments was not to replicate natural environments, it is clear from these results and others that both initial community composition and physical structure are likely both substantive controls on the composition of mixed environmental communities, as are intraspecific competition, mutualism, and other forms of organismal interaction (Konopka et al., 2015).

The parallelized enrichment strategy we employ here provides a framework to capture some of the underlying and potentially novel interactions between microbial community members found in soil and water in a high-throughput manner. This work also has the potential to inform the synthetic design of microbial communities, an area of considerable interest (Shou et al., 2007; Mee et al., 2014; Stenuit and Agathos, 2015). Current work in our laboratory is focused on testing the stability of these “minimal communities” in continuous culture systems with the hope of creating hybrid synthetic communities that are sourced directly from specific environments but streamlined in the laboratory. The isolation of a consistently reoccurring generalist provides an anchor within the community for genetic engineering. A variety of genetic tools are available for *Pseudomonas* and other taxa seen here, providing a potential platform for customization of minimal communities for industrial and biomedical applications. Moving forward, as desire increases to engineer microbial communities either by strain selection or outright genetic manipulation (Johnson et al., 2012; Lindemann et al., 2016), the use of highly parallelized microbial enrichments promises to provide novel insights that will allow us to predict with greater accuracy the interactions that contribute to emergent properties of microbial communities, such as community stability.

## Author contributions

DA, TF, and KK: designed the project; TF: collected samples and conducted the experiments; JK: extracted DNA and prepared sequencing libraries; SG and SO: carried out DNA sequencing; TF and DA: analyzed the data and wrote the manuscript; All authors reviewed and approved the final manuscript.

### Conflict of interest statement

The authors declare that the research was conducted in the absence of any commercial or financial relationships that could be construed as a potential conflict of interest.

## Footnotes

^1^ANOSIM was also performed using the Bray-Curtis similarity coefficient and exhibited similar trends (data not shown).
