# Supplementary material for: Parallelized, Aerobic, Single Carbon-Source Enrichments from Different Natural Environments Contain Divergent Microbial Communities
Source: Front Microbiol. 2017 Nov 28;8:2321. doi: 10.3389/fmicb.2017.02321 (PMC5712364; doi:10.3389/fmicb.2017.02321)
Supplement: Supplementary file 1 [file Image1.PDF]

Supplementary Material

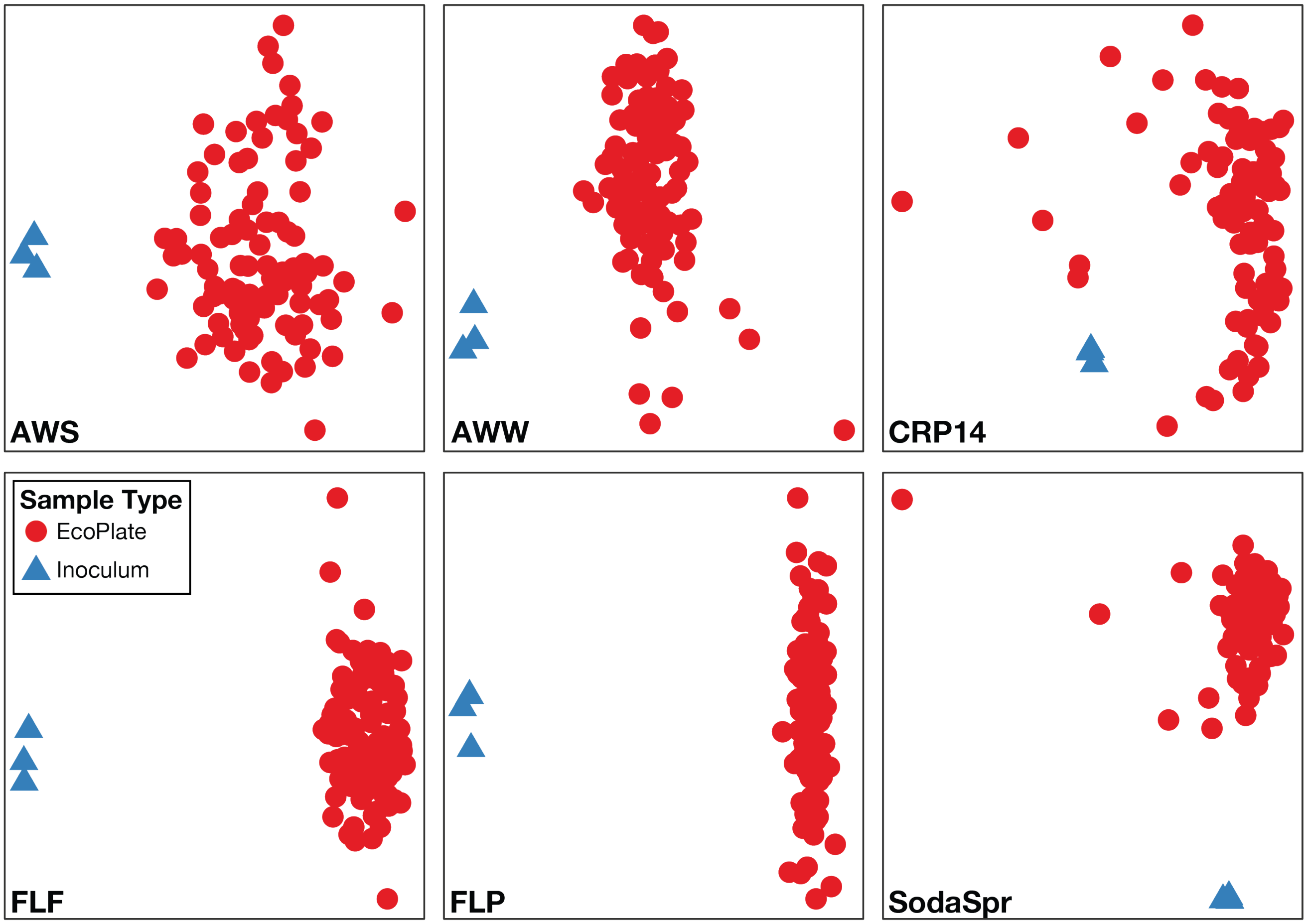

Figure S1. NMDS plots highlighting the difference in microbial community structure between environmental inocula (blue) and EcoPlate enrichments (red). Each individual plot includes all samples from a particular environment: freshwater wetland soil (AWS), freshwater wetland water (AWW), tropical forest soil (CRP14), temperate forest soil (FLF), temperate prairie soil (FLF), subalpine forest soil (SodaSpr).

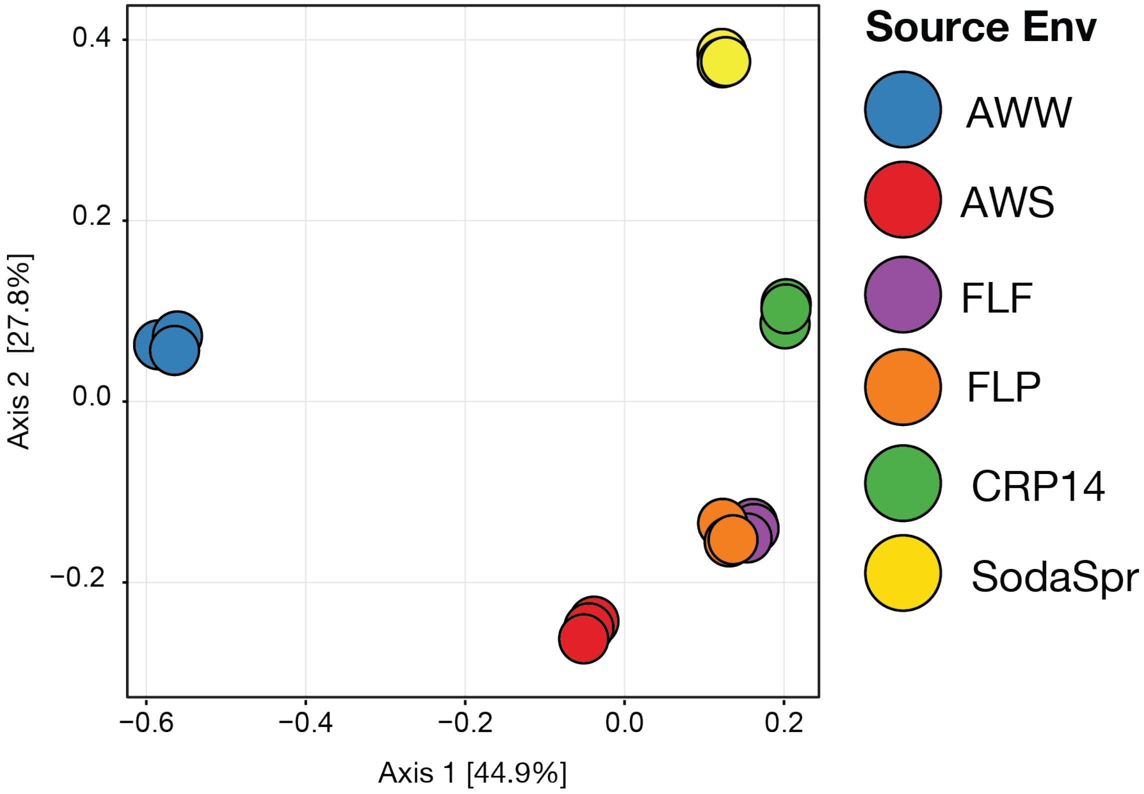

Figure S2. Principal coordinates (PCoA) plot of the differences between triplicate samples of the environmental inocula used in substrate array experiments: freshwater wetland soil (AWS), freshwater wetland water (AWW), tropical forest soil (CRP14), temperate forest soil (FLF), temperate prairie soil (FLF), subalpine forest soil (SodaSpr). Primary source of variation along Axis 1 is between soil communities and planktonic cells from wetland surface water (AWW). Additional variation along axis two appears to differentiate between microbial communities in Midwestern soil (AWS, FLF, and FLP) compared to subalpine (SodaSpr) and tropical (CRP14) soil.
